# Infection prevention and control measures during the COVID-19 pandemic and airborne tuberculosis transmission during primary care visits in South Africa

**DOI:** 10.1016/j.ijid.2025.107921

**Published:** 2025-05-06

**Authors:** Nicolas Banholzer, Keren Middelkoop, Remo Schmutz, Juane Leukes, Kathrin Zürcher, Matthias Egger, Robin Wood, Lukas Fenner

**Affiliations:** 1 Institute of Social and Preventive Medicine, University of Bern, Bern, Switzerland; 2 Institute of Infectious Disease and Molecular Medicine, University of Cape Town, Cape Town, South Africa; 3 Desmond Tutu HIV Centre, Department of Medicine, University of Cape Town, Cape Town, South Africa; 4 Centre for Infectious Disease Epidemiology & Research, School of Public Health & Family Medicine, University of Cape Town, Cape Town, South Africa; 5 Population Health Sciences, Bristol Medical School, University of Bristol, Bristol, UK; 6 Desmond Tutu Health Foundation, Cape Town, South Africa

**Keywords:** Airborne transmission, Tuberculosis, Carbon dioxide, Infection prevention and control, Genomic DNA, Healthcare facilities

## Abstract

**Background:**

Tuberculosis (TB) transmission in crowded health care settings poses considerable risks in high-burden regions. We assessed how COVID-19 pandemic-related infection prevention and control (IPC) measures might impact TB transmission in a South African primary care clinic.

**Methods:**

In 2019 (prepandemic) and 2021 (pandemic), we collected clinical data, patient tracking data (person-time and spatial density), indoor CO_2_ levels, and concentrations of *Mycobacterium tuberculosis* (*Mtb*) DNA in bio-aerosol samples. We estimated the risk of *Mtb* transmission during a 1-hour visit based on ventilation rate and duration of exposure.

**Results:**

During the pandemic, clinics were less crowded, with lower mean person-time per day (209 vs 258 hours; *P <* 0.001). TB prevalence among patients also declined (1.1% vs 4.7%; *P* = 0.052). Environmental indicators suggested improved air quality, with lower CO_2_ levels (555 vs 856 parts-per-million; *P <* 0.001) and higher ventilation rates (15.8 vs 6.3 air changes per hour; *P <* 0.001). These changes corresponded with a reduction in modeled *Mtb* transmission risk (0.04% vs 1.36%; *P* = 0.046). Airborne *Mtb* DNA was detected in both periods.

**Conclusion:**

Pandemic-related IPC measures to control COVID-19 transmission were rigorously implemented and likely reduced transmission of airborne respiratory infections, supporting their continued implementation in healthcare settings postpandemic.

## Introduction

Tuberculosis (TB), caused by the bacterium *Mycobacterium tuberculosis* (*Mtb*), is a strictly airborne pathogen [1] and, until the COVID-19 pandemic, was the leading cause of death from all infectious diseases worldwide. It is increasingly being recognized that other respiratory infections, such as SARS-CoV-2 are also primarily transmitted by aerosols [1]. Airborne transmission is more likely in crowded indoor settings such as healthcare facilities.

Infection prevention and control (IPC) measures play a key role in reducing airborne transmission in healthcare settings [2]. In 2019, we estimated the risk of *Mtb* transmission in a South African clinic [3] and repeated the study in 2021 during the COVID-19 pandemic when several IPC measures were implemented. We compared patient and environmental data before and during the COVID-19 pandemic to evaluate IPC interventions.

## Methods

For 6 weeks in July/August 2019 (pre-COVID-19) and October/November 2021 (during the COVID-19-pandemic), we conducted studies in the waiting room of a primary care clinic in Cape Town, South Africa [3,4], using the introduction of pandemic-related IPC measures as a natural experiment. The clinic provides TB/HIV, pediatric, and other services 7 am-4 pm, Monday-Friday, and is situated within a large settlement of formal and semiformal housing with a high TB/HIV burden. Ventilation rate, person-time in clinic, and *Mtb* DNA concentrations were the outcomes, and the estimated transmission risk is the modeled impact. Several IPC measures were implemented by public health authorities during the pandemic (Supplementary Table S1), including face masks for all patients and staff, physical distancing, increased natural ventilation, additional outdoor waiting areas, and patient triage. Due to holidays, staff training, and scheduled electricity outages, observation days differed in 2019 and 2021 (Supplementary Figure S1).

Measurements were identical during the two study phases. We used optical sensors to analyze crowding in the waiting room (Xovis, Switzerland) and measured indoor CO_2_ levels in parts-per-million (ppm) (CL11, Rotronic, Switzerland). Air was sampled using mobile bioaerosol samplers (Lockheed-Martin Integrated Systems, USA), and the number of *Mtb* genomes determined from polyester-felt filters using highly sensitive droplet digital PCR (ddPCR) [3,5]. Anonymized clinical data were obtained from the electronic clinic database (including TB/HIV diagnostics).

We calculated person-time (hours), ventilation rates (air changes per hour) [6], and *Mtb* concentrations (log DNA copies/*μ*L per sampling hour), overall and by daytime (morning 8 am-12 pm, afternoon 12 pm-4 pm). The risk of *Mtb* transmission (%) was modeled using the Wells–Riley equation [6], based on empirical and published parameters (Supplementary Table S2, Text S1). We present means ± standard deviations throughout. We used generalized linear regression models adjusted for daytime to compare empirical outcomes and modeled transmission risk between study years. Analyses were conducted in R (version 4.2.1).

## Results

**Figure 1.**
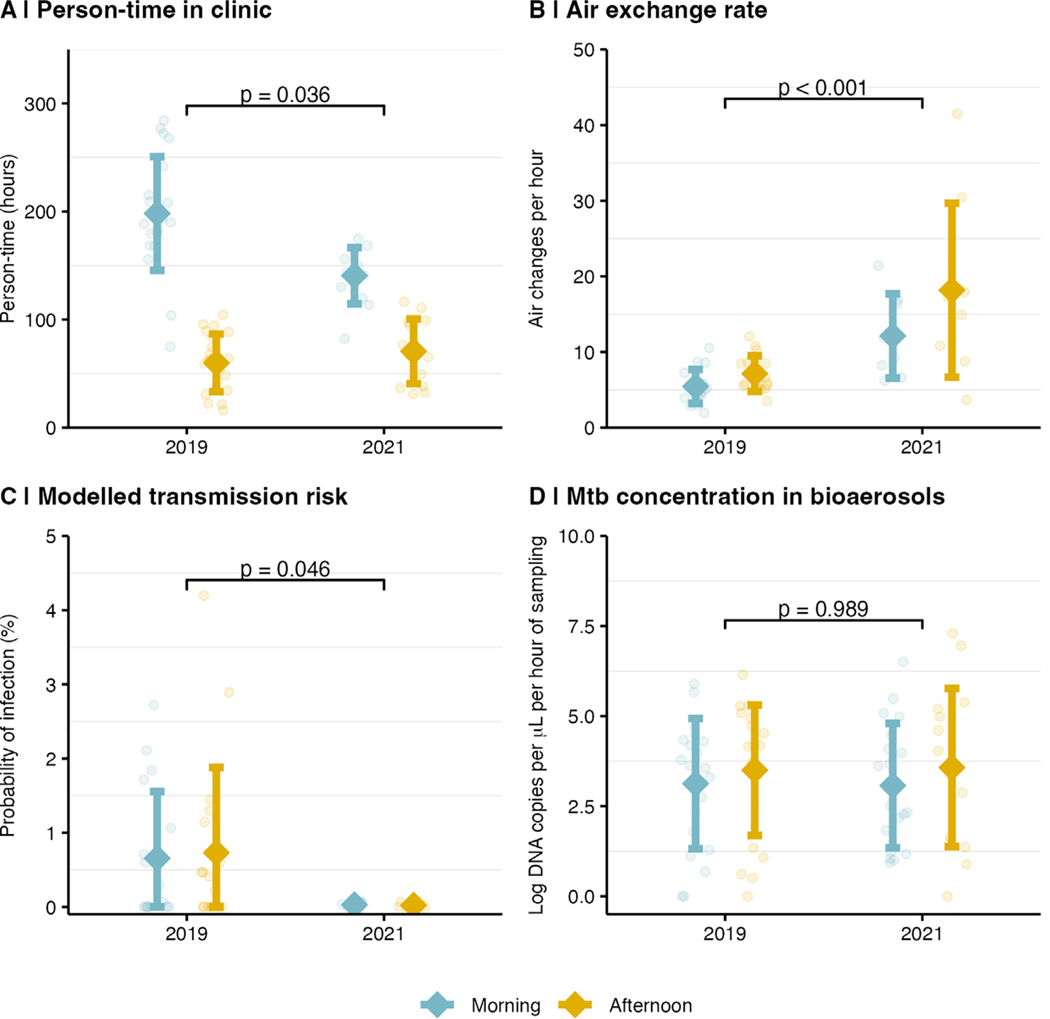
Comparison of patient and environmental data during (with implemented infection prevention and control [IPC] measures) and before the COVID-19 pandemic, by daytime. Clinic occupancy (person-time, a), air exchange rate (b), modeled TB transmission risk using the Wells–Riley equation (c), and *Mycobacterium tuberculosis (Mtb)* DNA concentrations (copies/μL) in the air (d). For each variable, the mean is shown as a dot, the ± standard deviation as an error bar, and individual observations are shown as grey jittered dots. Statistical comparisons are based on two-sample *t*-tests.

**Figure 2.**
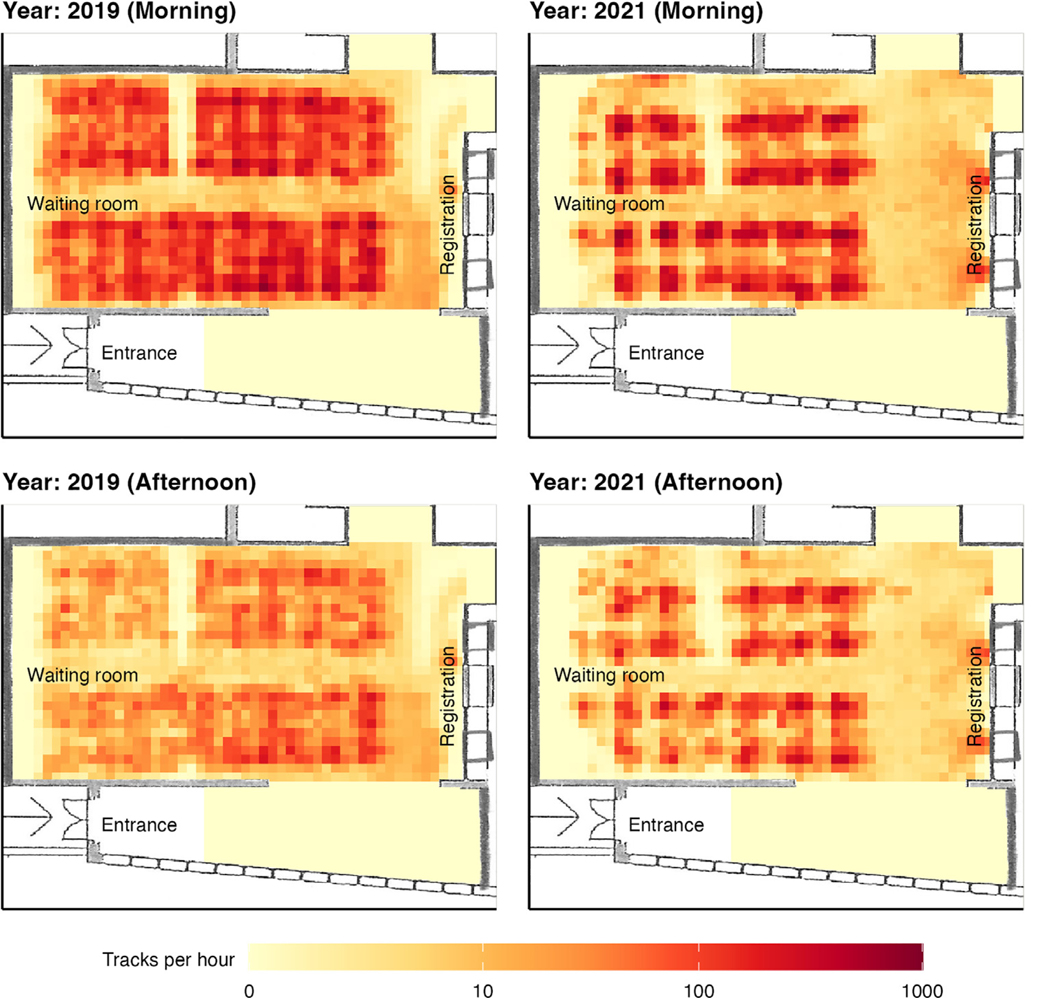
Comparison of crowding in the waiting room during (with IPC measures implemented) and before the COVID-19 pandemic, including physical distancing and restricted patient flows. The density of people in the clinic waiting room was measured using optical sensors.

Person-time, ventilation rate, *Mtb* concentrations, and modeled transmission risk are shown in Figure 1 and Supplementary Table S3 for the periods before and during the pandemic (with IPC). Compared to the pre-COVID-19 period, daily person-time in the clinic was lower during the pandemic (209 vs 258 person-hours, *P* = 0.036); the crowding of visitors and patients was also reduced, with low-density areas on benches due to physical distancing (Figure 2). Daily maximum CO_2_ levels were lower (554 vs 856 ppm, *P <* 0.001; Supplementary Table S3) and air exchange rates higher during the pandemic (15.8 vs 6.3/hour, *P <* 0.001) than before. *Mtb* genomic DNA in bio-aerosols was detected at comparable concentrations in both periods, but ddPCR results varied considerably between sampling days (Supplementary Figure S2). The modeled risk of infection for a 1-hour exposure time was lower during the pandemic (0.04% vs 1.36%, odds ratio 0.36, 95%-confidence interval 0.13–0.98, *P* = 0.046). The difference persisted under different modeling scenarios (Supplementary Figure S3).

The TB prevalence among clinic patients appeared to be lower during the pandemic (1.1% vs 4.7%, *P* = 0.052; Supplementary Table S3). TB case-to-suspected ratio and HIV positivity were similar in both study periods, but TB patients during the pandemic tended to have higher bacterial loads (Supplementary Figure S4). Due to the different sampling times in 2019 and 2021, differences in the climatic conditions were noted (Supplementary Figure S5).

## Discussion

At a South African clinic, we assessed the impact of IPC measures introduced during the COVID-19 pandemic to mitigate SARS-CoV-2 transmission, comparing environmental, patient, and molecular data collected before and during the pandemic. IPC measures increased natural ventilation, reduced crowding and clinic visit time, and lowered the modeled risk of transmission, indicating rigorous implementation and potential effectiveness.

Environmental factors are crucial in controlling respiratory infections and preventing airborne transmission. TB is recognized as a strictly airborne infectious disease, and a role of aerosol transmission in the spread of SARS-CoV-2 is now well-established [7]. CO_2_ measurements can be used to estimate the risk of airborne transmission using the Wells–Riley equation [6], an epidemiological model that incorporates ventilation rates and exposure time to infectious doses. Research has consistently shown that poorly ventilated indoor spaces facilitate transmission of respiratory infections [8–10].

We found that a combination of IPC measures increased natural ventilation and reduced crowding, leading to a lower risk of airborne transmission. Simple actions like opening doors and windows can substantially improve ventilation in resource-limited settings with a high TB burden. Higher-income countries should also invest in improved building designs and ventilation systems. Environmental IPC measures can be purposefully combined with clinic-based administrative measures (e.g., respiratory separation) and respiratory protection (e.g., the use of face masks), in line with WHO’s three-level hierarchy of TB IPC recommendations [2]. Our evaluation of these interventions in a clinic setting are in line with findings from a school-based study (9), strengthening the evidence for their effectiveness in reducing transmission risk in crowded settings.

We detected airborne *Mtb* DNA in both periods. Airborne *Mtb* genomic DNA has previously been detected in South African clinics and school classrooms, demonstrating the risk of exposure to air-borne *Mtb* and potential transmission [11]. Differences in *Mtb* concentrations before and during the pandemic were small and out-weighed by the large variation in ddPCR results.

The COVID-19 pandemic raised concerns about possible interruptions of clinic services, including TB/HIV services. A study from South Africa found a decrease in TB diagnoses and treatment initiations during the pandemic [12]. Similarly, we observed that the number of TB patients during the pandemic was lower and that patients tended to have more advanced disease. Efforts are needed to maintain access to essential TB services in high-burden countries during pandemics [13]. By minimizing the risk of transmission, IPC measures can help sustain uninterrupted delivery of clinical services, even during public health crises.

Our study has limitations. First, while the observed reduction in crowding may partly reflect the overall decrease in clinic attendance during the pandemic, other factors—such as ventilation rates—are less dependent on patient volume. Second, the ddPCR assay detecting *Mtb* genomic DNA cannot distinguish between viable and noninfectious bacilli [3,5,11]. Additionally, data collection was affected by international travel restrictions during the pandemic, resulting in slightly different study periods. This may have introduced minor seasonal effects, although the impact of temperature and humidity on transmission is complex and not fully understood.

In conclusion, we demonstrated that IPC interventions were rigorously implemented during the pandemic in a South African clinic. These practical measures likely reduced transmission of air-borne respiratory infections, including TB and SARS-CoV-2, and should be sustained beyond the pandemic, especially in clinics in high TB-burden countries.

## Supplementary Material

MMC1

Supplementary material associated with this article can be found, in the online version, at doi:10.1016/j.ijid.2025.107921.
