# Supplementary material for: Infection prevention and control measures during the COVID-19 pandemic and airborne tuberculosis transmission during primary care visits in South Africa
Source: Int J Infect Dis. Author manuscript; Available in PMC 2025 Jul 21. (PMC12277576; doi:10.1016/j.ijid.2025.107921)
Supplement: MMC1 [file NIHMS2090796-supplement-MMC1.docx]

# SUPPLEMENTARY INFORMATION

**Supplementary Table S1. Infection prevention and control (IPC) measures introduced during the COVID-19 pandemic in a primary care clinic in South Africa.**

| **IPC** | **Description** |
| --- | --- |
| **Physical distancing** | Physical distancing was enforced outside the clinic while waiting and inside the registration area and waiting room (including on benches). |
| **Masks** | Face masks (at least surgical or community masks) were required to be worn by all clinic visitors and staff, except for children. |
| **Restricted patient flow** | Patients were given fixed appointments to avoid overcrowding and reduce waiting times in the clinic. They were asked to arrive no more than one hour before their appointment. Otherwise, they were asked to wait outside the clinic. |
| **Patient triage** | Clinic visitors and patients with respiratory symptoms were screened outside the clinic before entering. |
| **Active ventilation** | Windows and doors in the clinic were opened throughout the day to actively increase natural ventilation. |

**Supplementary Table S2. Assumptions for modeling *Mycobacterium tuberculosis* transmission risk using the Wells-Riley equation.**

| **Modeling parameter** | **Assumption** |
| --- | --- |
| **Number of diagnosed TB patients visiting the clinic, n** | See Supplementary Table 1 |
| **Ventilation rate (air exchange rate), h^-1^** | See Supplementary Table 1 |
| **Infectious dose (quanta generation rate), h^-1^** | 2 quanta |
| **Breathing rate, m^3^/h** | 0.5 |
| **CO_2_ generation rate, L/s** | 0.004 |
| **Exposure time, h** | 1 |

h, hours

**Supplementary Table S3. Comparison of patient, environmental, and molecular data during and before the COVID-19 pandemic.** Statistical comparisons are based on daytime-adjusted generalized linear regression models.

| **Variable**  Mean (SD) | **Pre-pandemic (2019)** | **Pandemic (2021)** | **p-value** |
| --- | --- | --- | --- |
| **Patient data** |  |  |  |
| Person-time, h | 258 (75) | 209 (53) | 0.036 |
| Registered patients, per day | 345 (242) | 114 (44) | <0.001 |
| Diagnosed TB patients, per day | 11 (11) | 1 (1) | <0.001 |
| Prevalence of TB ^1^, % | 4.7 (6.5) | 1.1 (0.8) | 0.052 |
| **Environmental data** |  |  |  |
| Daily maximum CO_2_, ppm | 856 (207) | 555 (82) | <0.001 |
| Air exchange rate ^2^, h^-1^ | 6.3 (2.4) | 15.8 (11.0) | <0.001 |
| **Molecular data** |  |  |  |
| *Mtb* concentration in the air, copies / µl, overall | 3.8 (1.2) | 3.8 (1.8) | 0.99 |
| **Modeled transmission** |  |  |  |
| Risk of infection ^3^, % | 1.36 (1.44) | 0.04 (0.03) | 0.046 |

h, hours; IQR, interquartile range; *Mtb*, *Mycobacterium tuberculosis*; ppm, particles per minute; SD, standard deviation; TB, tuberculosis

^1^ We defined TB prevalence as the proportion of the daily number of diagnosed TB patients visiting the clinic who had been on TB treatment for less than 28 days among all patients visiting the clinic during that day (prevalence of potentially infectious persons).

^2^ Air exchange rate (ventilation rate) was calculated from time-varying CO_2_ and clinic occupancy using a transient mass balance model (see Supplementary Text S1 for details).

^3^ One hour of exposure (e.g. a one-hour visit to the clinic).

We used Gaussian models for person-time, CO_2_, air exchange rate, and *Mtb* concentration, Poisson models for registered patients and diagnosed patients, and Beta models with a logit link for TB prevalence and risk of infection.

**Supplementary Figure S1. Data availability by type, study day and year.** Environmental: CO_2_, temperature, humidity; Bio-aerosols: *Mtb* DNA in air; Tracking: person-time, density of people. Clinical data were available for all days.

**
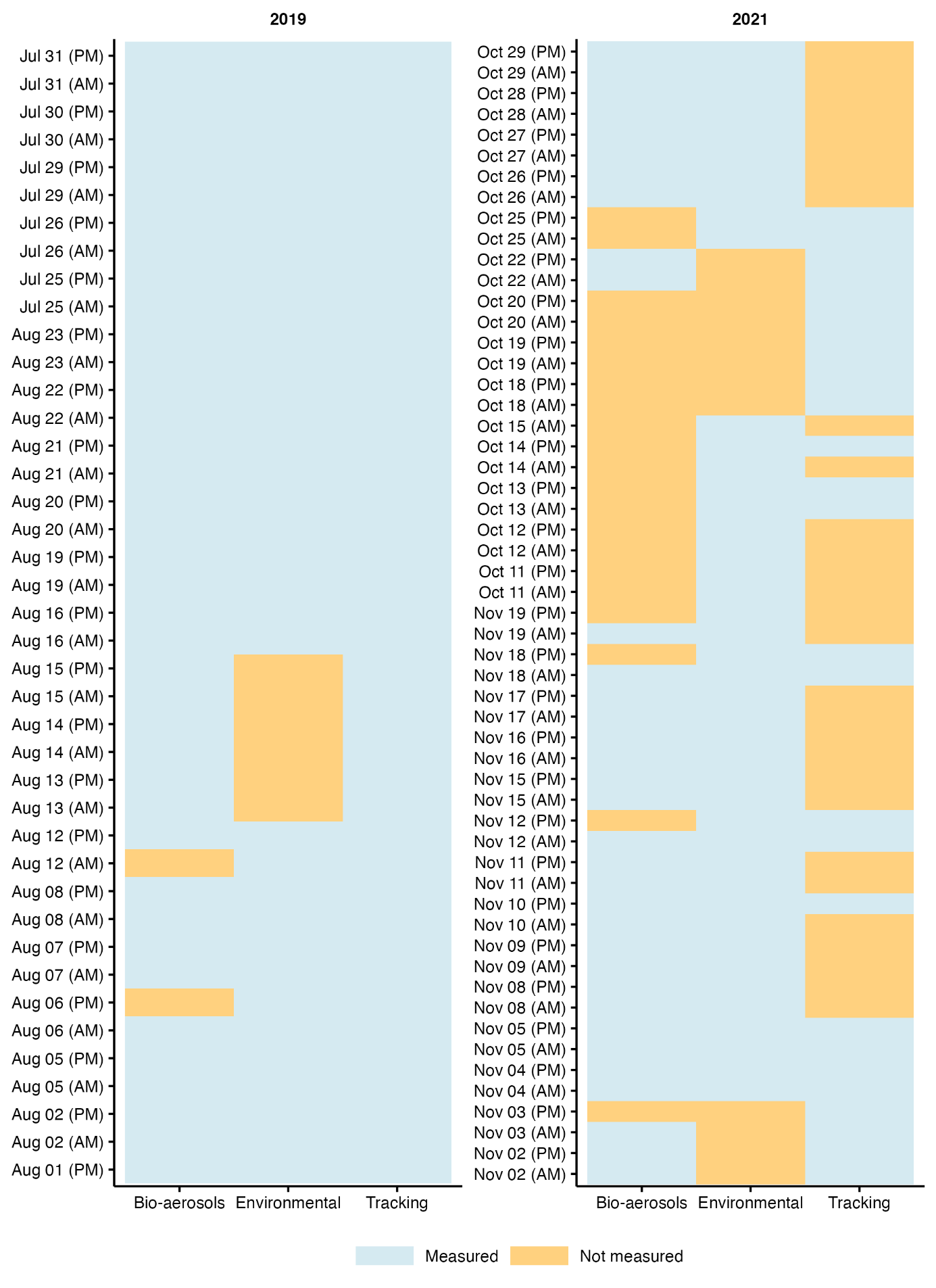
**

**Supplementary Figure S2. Coefficients of variation (standard deviation divided by the mean).** See also Figure 1.

**
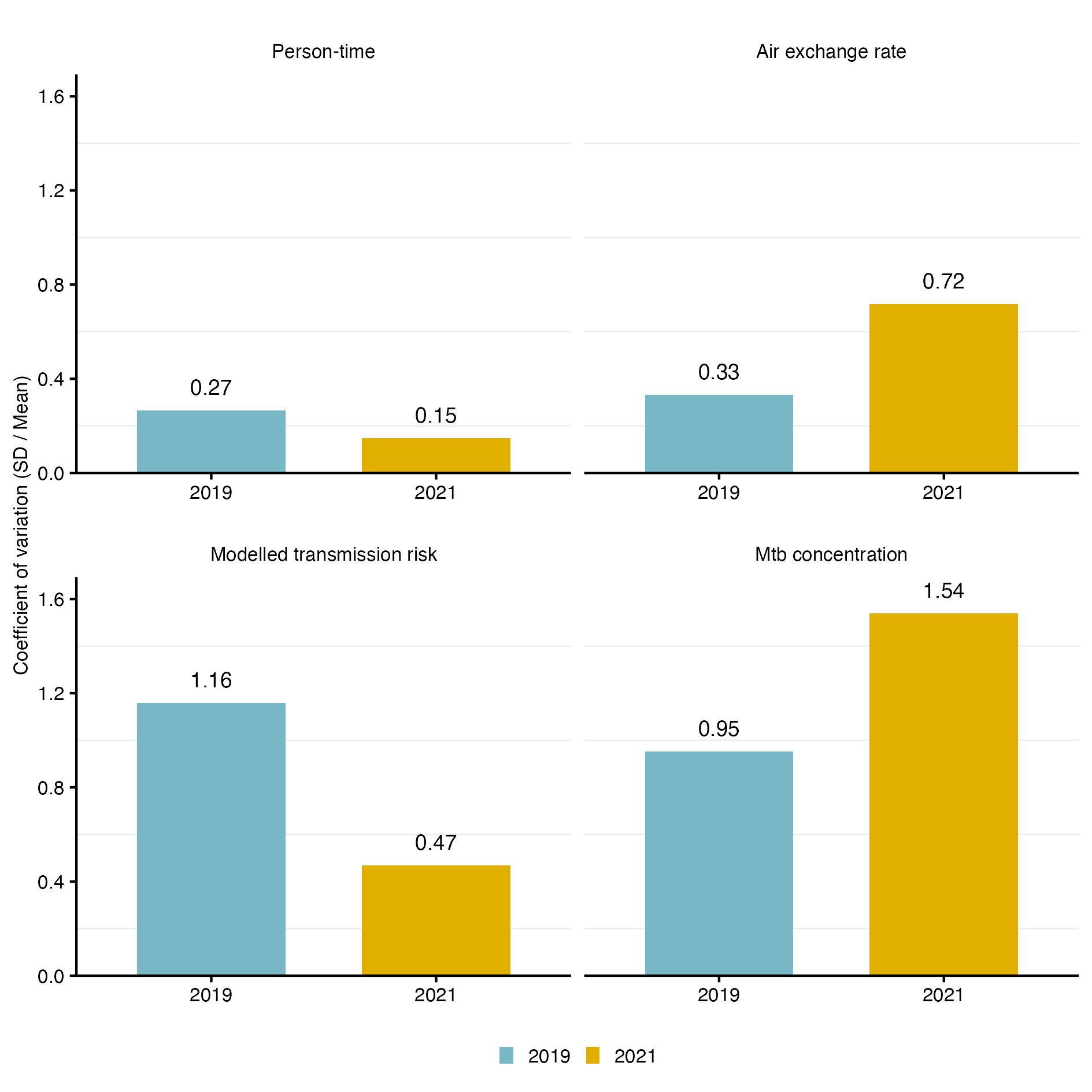
**

**Supplementary Figure S3: Modeled transmission (risk of infection) under two different scenarios. A:** Assuming the same TB prevalence and exposure time in the two study years (impact of different ventilation conditions). **B:** Assuming the same TB prevalence, but different exposure times according to the changes in person-time in the clinic in the two study years (impact of different ventilation conditions and clinic occupancy). For each variable, the mean is shown as a dot, the ± standard deviation as an error bar, and individual observations are shown as grey jittered dots. Statistical comparisons are based on two-sample t-tests.


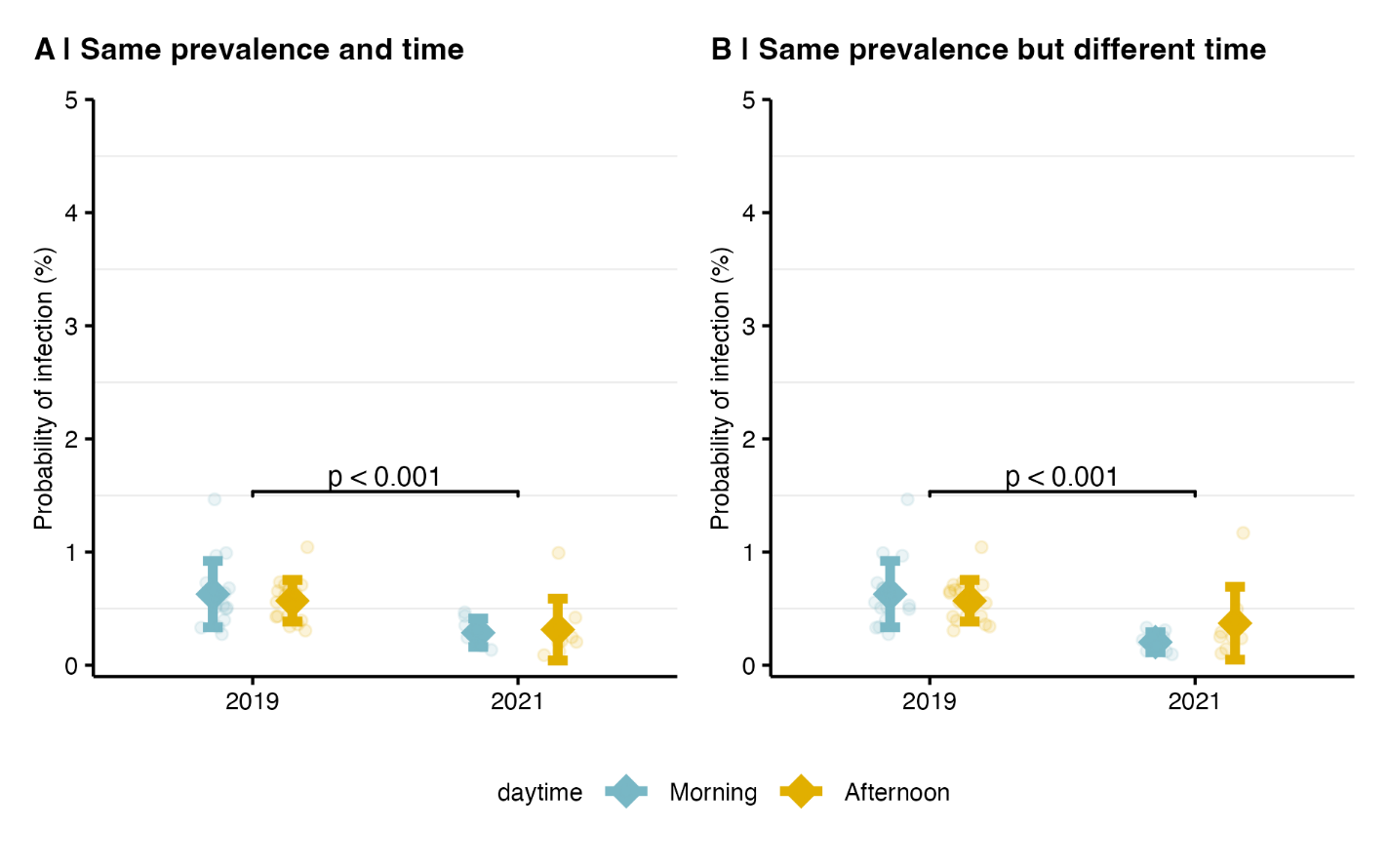


**Supplementary Figure S4. Comparison of TB patient characteristics during and before the COVID-19 pandemic.** TB case-to-suspect ratio was defined as GeneXpert MTB/RIF-positive patients among all patients with a GeneXpert test result and bacterial load as sputum smear microscopy grading (limited to individuals with a smear microscopy result).


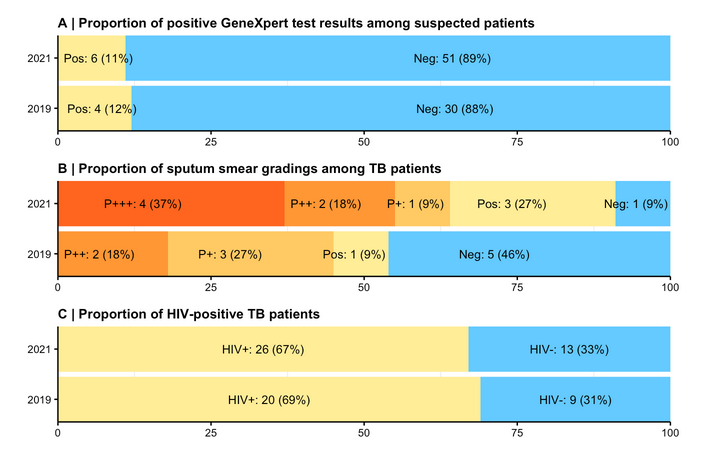


**Supplementary Figure S5: Comparison of the climatic conditions (temperature and relative humidity) before COVID-19 (July/August 2019) and during the pandemic (October/November 2021).** For each variable, the mean is shown as a dot, the ± standard deviation as an error bar, and individual observations are shown as grey jittered dots. Statistical comparisons are based on two-sample t-tests.

**
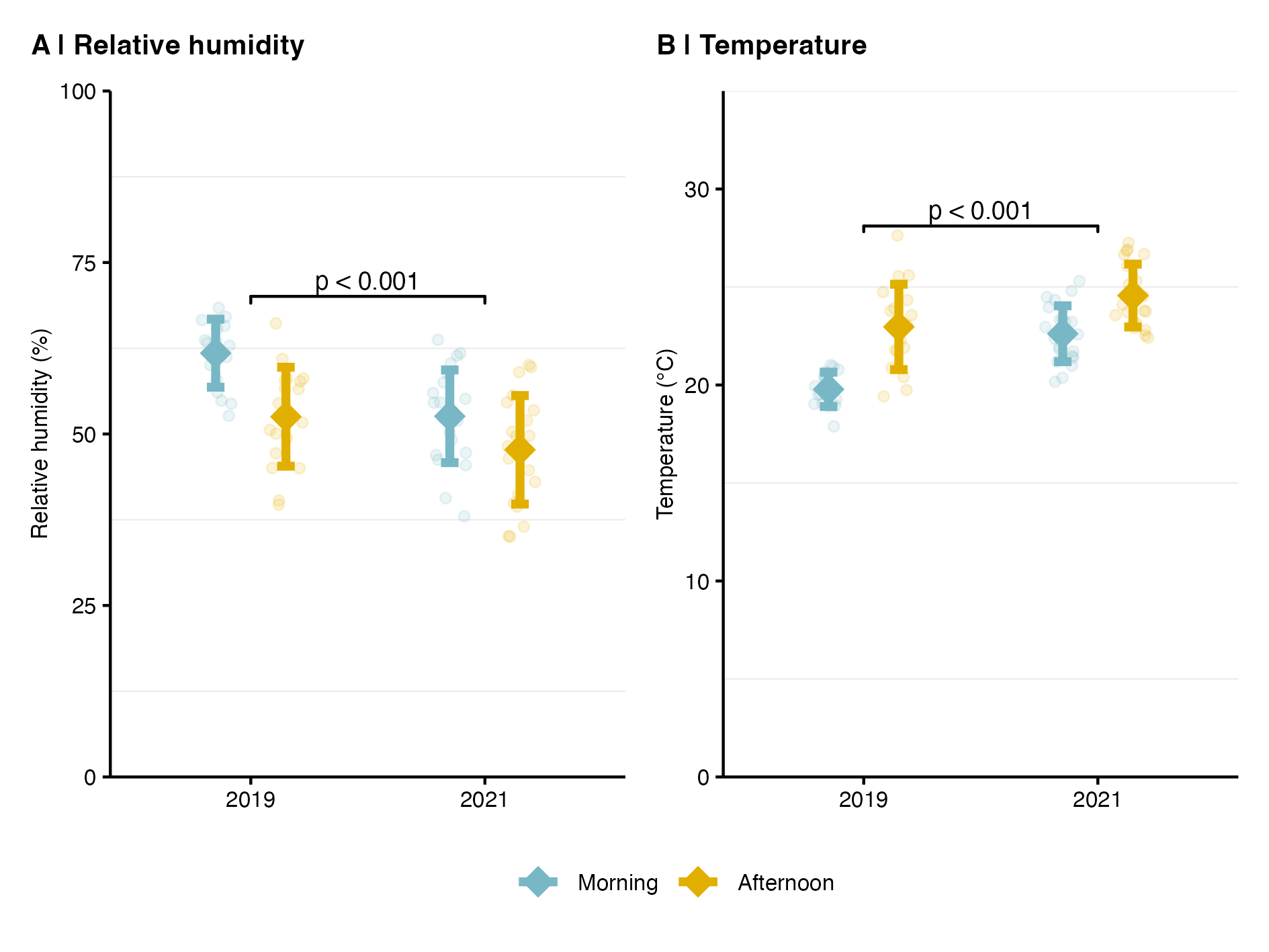
**

**Supplementary Text S1: Description of the methods used to model the tuberculosis (TB) transmission risk.**

We modeled tuberculosis transmission (risk of infection) in the clinic using the Wells-Riley equation, which estimates the risk (probability P in %) as:

$P =\frac{C}{S} = 1 -\exp\left( -\frac{Ipqt}{Q} \right)$,

where $C$ is the number of diseased cases, $S$ is the number of susceptible cases, $I$ is the number of infectious persons in the indoor space, $p$ is the breathing rate per person (m^3^ h^-1^), $q$ is the quantum (infectious dose) generation rate (quanta h^-1^), $t$ is the exposure time (h), and $Q$ is the ventilation rate (m^3^ h^-1^). The probability of infection is estimated with a Poisson relation, taking into account the stochastic behavior of airborne infection, where one quantum corresponds to a 67% risk of infection.

Based on the literature, we assumed a constant breathing rate of 0.5 m^3^ h^-1^ [1] and a quanta generation rate of 2 quanta h^-1^ [2]. The number of infectious persons in the clinic corresponded to the daily number of diagnosed TB patients visiting the clinic in the morning and afternoon. Ventilation rate (and air exchange rate) was also calculated by daytime (morning: 8 am to 12 pm, afternoon: 12 pm to 4 pm) from time-varying CO_2_ and clinic occupancy using a transient mass balance model [3], assuming a CO_2_ generation rate of 0.004 L/s [4]. The volume of the clinic was 174m^3^. In the main analysis we considered an exposure time of one hour for susceptible individuals visiting the clinic. The modeling parameters are summarised in Supplementary Table S2.

As an additional analysis, we modeled the risk of infection in two additional scenarios to disentangle the impact of intervention and prevention control (IPC) measures and lower TB prevalence (Supplementary Figure S3). In both scenarios, we replaced the year-specific TB prevalence among clinic visitors with a fixed TB prevalence corresponding to the mean number of diagnosed TB patients in 2019 in the morning (5.1 patients) and afternoon (6.4 patients). In scenario (A), the TB prevalence was the same in both study years and we also kept the exposure time at one hour for both study years. In scenario (B), we kept the same TB prevalence but replaced the exposure time in 2021 with the mean ratio of person-time in the clinic between 2021 and 2019 (morning: 0.70h, afternoon: 1.18h). Scenario (A) isolates the impact of different ventilation conditions, while scenario (B) takes into account changes in both ventilation conditions and clinic occupancy.

**References:**

1. Banholzer N, Schmutz R, Middelkoop K, Hella J, Egger M, Wood R, et al. Airborne transmission risks of tuberculosis and COVID-19 in schools in South Africa, Switzerland, and Tanzania: Modeling of environmental data. PLOS Glob Public Health. 2024;4: e0002800. doi:10.1371/journal.pgph.0002800

2. Assessment UENC for E. Measurement of breathing rate and volume in routinely performed daily activities [final report]. 15 Mar 2009 [cited 26 Feb 2025]. Available: https://hero.epa.gov/hero/index.cfm/reference/details/reference_id/77086

3. Batterman S. Review and extension of CO₂-based methods to determine ventilation rates with application to school classrooms. Int J Environ Res Public Health. 2017;14: 145. doi:10.3390/ijerph14020145

4. Persily A, de Jonge L. Carbon dioxide generation rates for building occupants. Indoor Air. 2017;27: 868–879. doi:10.1111/ina.12383
